# Genomic mechanisms for cold tolerance and production of exopolysaccharides in the Arctic cyanobacterium *Phormidesmis priestleyi* BC1401

**DOI:** 10.1186/s12864-016-2846-4

**Published:** 2016-08-02

**Authors:** Nathan A. M. Chrismas, Gary Barker, Alexandre M. Anesio, Patricia Sánchez-Baracaldo

**Affiliations:** 1 Bristol Glaciology Centre, School of Geographical Sciences, University of Bristol, Bristol, BS8 1SS UK; 2 Cereal Genomics, School of Biological Sciences, University of Bristol, Bristol, BS8 1SS UK

**Keywords:** Cyanobacteria, Cryosphere, Cryoconite, Cold-adaptation, Exopolysaccharides, Biofilms, ABC-transporters, c-di-GMP

## Abstract

**Background:**

Cyanobacteria are major primary producers in extreme cold ecosystems. Many lineages of cyanobacteria thrive in these harsh environments, but it is not fully understood how they survive in these conditions and whether they have evolved specific mechanisms of cold adaptation. *Phormidesmis priestleyi* is a cyanobacterium found throughout the cold biosphere (Arctic, Antarctic and alpine habitats). Genome sequencing of *P. priestleyi* BC1401, an isolate from a cryoconite hole on the Greenland Ice Sheet, has allowed for the examination of genes involved in cold shock response and production of extracellular polymeric substances (EPS). EPSs likely enable cyanobacteria to buffer the effects of extreme cold and by identifying mechanisms for EPS production in *P. priestleyi* BC1401 this study lays the way for investigating transcription and regulation of EPS production in an ecologically important cold tolerant cyanobacterium.

**Results:**

We sequenced the draft genome of *P. priestleyi* BC1401 and implemented a new de Bruijn graph visualisation approach combined with BLAST analysis to separate cyanobacterial contigs from a simple metagenome generated from non-axenic cultures. Comparison of known cold adaptation genes in *P. priestleyi* BC1401 with three relatives from other environments revealed no clear differences between lineages. Genes involved in EPS biosynthesis were identified from the Wzy- and ABC-dependent pathways. The numbers of genes involved in cell wall and membrane biogenesis in *P. priestleyi* BC1401 were typical relative to the genome size. A gene cluster implicated in biofilm formation was found homologous to the Wps system, although the intracellular signalling pathways by which this could be regulated remain unclear.

**Conclusions:**

Results show that the genomic characteristics and complement of known cold shock genes in *P. priestleyi* BC1401 are comparable to related lineages from a wide variety of habitats, although as yet uncharacterised cold shock genes in this organism may still exist. EPS production by *P. priestleyi* BC1401 likely contributes to its ability to survive efficiently in cold environments, yet this mechanism is widely distributed throughout the cyanobacterial phylum. Discovering how these EPS related mechanisms are regulated may help explain why *P. priestleyi* BC1401 is so successful in cold environments where related lineages are not.

**Electronic supplementary material:**

The online version of this article (doi:10.1186/s12864-016-2846-4) contains supplementary material, which is available to authorized users.

## Background

Cyanobacteria are photosynthetic prokaryotes that have thrived on our planet for at least 2.33–2.4 Ga [1, 2]. In the cryosphere cyanobacteria are major primary producers found in many types of habitats (e.g., lakes, lithic substrates and cryoconite [3]) where potential environmental pressures include exposure to high levels of ultraviolet radiation during the summer, complete absence of light during the winter at the Poles, limited nutrient availability, rapid freeze-thaw cycles and low water activity [4].

Microorganisms (including bacteria, archaea and unicellular eukaryotes) have numerous adaptations that enable them to survive at low temperatures. Generic cold-shock mechanisms exist (many of which have been characterised in mesophiles like *Escherichia coli*) that are responsible for maintaining cell membrane fluidity, destabilising nucleic acid secondary structures and helping to maintain chromosome structure in cold conditions (reviewed in Barria et al. [5]). Other mechanisms that have been proposed include the production of exopolysaccharides (EPSs), which play a major role in protection from extreme cold environments (e.g., Bacteria: *Colwellia psychrerythraea* 34H [6], *Pseudomonas* sp. ID1 [7] and *Pseudoalteromonas* sp. SM20310 [8]; Archaea: *Methanococcoides burtonii* and *Halorubrum lacusprofundi* [9]; Diatoms: *Synedropsis* sp., *Fragilariopsis curta* and *F. Cylindrus* [10]. In cyanobacteria, EPSs are known to enable desiccation resistance in arid habitats [11–13] and contribute to biofilm formation [14, 15]. In cold environments EPSs may have a role in freeze-thaw tolerance [12, 13]. EPSs likely provide a site for the localisation of ultraviolet protective compounds such as scytonemin and mycosporine-like amino acids (MAAs) and allow for the scavenging of metal cations in oligotrophic conditions [16]. EPSs also serve as a carbon source for the microbial web [17–19].

While all of these mechanisms can be found in the majority of prokaryotes, little is understood about how they might differ in cyanobacteria from cold environments compared to their temperate relatives. Many cyanobacteria commonly found in the cryosphere are also found in a variety of diverse habitats (e.g., deserts: *Chroococcidiopsis* [20] and *Nostoc* [21]; caves: *Chroococcidiopsis*, *Nostoc*, *Phormidium*, *Leptolyngbya* and *Pseudanabaena* [22]; soil crusts: *Phormidium, Leptolyngbya* and *Pseudanabaena* [23]) where their persistence is due to a non-specific resilience to extreme environments. Some lineages appear more localised to the cryosphere and as such may be more specialised to surviving in the cold. One such organism is the EPS producing, non-heterocystous filamentous cyanobacteria *Phormidesmis priestleyi*. Analysis of small subunit (SSU) rRNA gene sequences has shown that *P. priestleyi* can be found throughout the global cryosphere [24, 25]. Trait evolution analyses have predicted that *P. priestleyi* had a cold tolerant ancestor [25].

In the Antarctic, *P. priestleyi* commonly grows in de-glaciated regions where it is typically found associated with moving water and glacial run-off [26]. In the Arctic and Alpine regions *P. priestleyi* SSU rRNA gene sequences have been recovered from surface ice on glaciers in Svalbard [27], oligotrophic lakes in the Pyrenees [28], glaciers on the Tibetan Plateau [29] and meltwater lakes on ice shelves in the Canadian High Arctic [24]. On the Greenland Ice Sheet (GrIS) it can be found in cryoconite holes; melt water pools on the surface of glaciers formed by reduced local albedo that contain inorganic and organic particulate matter [30]. Cyanobacterial filaments constitute a large proportion of organic matter present in cryoconite granules and it is believed that cyanobacteria help to mediate the formation of cryoconite through the aggregation of particulate matter [31, 32] thus directly influencing the ‘biocryomorphology’ of ice surfaces [33].

In this study we sequenced the genome of *P. priestleyi* BC1401 using a recently developed de Bruijn graph assembly viewer to remove non-cyanobacterial sequences from the assembly. We used BLAST analyses to investigate the presence of known cold stress related genes in this and closely related genomes. We identified genes for putative mechanisms responsible for the regulation, production and export of polysaccharides using BLAST and Pfam domain searches. *P. priestleyi* BC1401 is the first published draft genome of a cyanobacterium isolated from the cryosphere.

## Methods

Cryoconite material was collected during the summer of 2014 from nearby the University of Utrecht’s S6 weather station on the GrIS (67°04′ N, 49°23′ W) ~1000 m above sea level and ~30 km from the ice margin, before being transported back to Bristol in a chilled container (~2–4 °C). In the laboratory, small amounts of cryoconite were transferred to petri dishes of sterile liquid BG-11 [34] and incubated at 4 °C for 4–6 weeks.

Axenic cultures have typically been used for the sequencing of single strains of cyanobacteria. However, several problems are associated with this approach. Firstly, certain strains may be reliant upon their commensal biota for successful growth e.g. due to utilisation of secondary metabolites [35]. Second, the process is labour intensive and hard to include within the timeframe of a small scale research program. Finally, cultures will undergo many generations during the process of growth and purification; this might result in significant molecular divergence of cultured strains from wild-type organisms. The methods shown here have allowed for the sequencing of a unialgal strain along with its commensal microbiota with the successful removal of contaminating (non-cyanobacteria) sequences during the assembly process. To obtain unialgal strains individual cyanobacterial filaments were separated using a microscope and glass needle, transferred to culture tubes containing sterile liquid BG-11, and incubated at 15 °C. After a further 4–6 weeks, a successful culture was transferred back to petri dishes of sterile liquid BG-11 and grown at 4 °C until sufficient biomass was available to extract gDNA. Harvested cells were stored in sterile 1.5 ml tubes at −20 °C.

### DNA extraction and sequencing

Extraction of DNA from cyanobacteria can often be inhibited by the presence of EPSs and thick cell walls. In order to address this issue we used a simple chemical/mechanical lysis method to rupture cells prior to extraction. Approximately 0.5 ml cellular material was defrosted and centrifuged at 13,000 G for 30 s in a 1.5 ml tube. The supernatant was removed and the pellet washed in 500 μl Milli-Q water. This process was repeated three times before the pellet was transferred to a clean 1.5 ml tube. The pellet was then re-suspended in 200 μl SoluLyse (Genlantis, San Diego, CA) (see Hall et al. [36]) and incubated at room temperature for 15 min. The entire contents of the tube were then transferred to a MO BIO (MO BIO Laboratories, Cambridge, UK) 0.7 mm bead beading tube and vortexed at full power for 5 min. gDNA was then extracted from the lysate using Machery-Nagel AXG20 (Machery-Nagel, Düren, Germany) gravity flow columns according to the manufacturers protocol (including the optional step of addition of lysozyme). Integrity of high molecular weight gDNA was assessed using gel imaging (1 % agarose gel for 1.5 h) and quantified using QUBIT (Invitrogen, Carlsbad, CA) assay before being sent for sequencing. Library preparation was done using the Illumina TruSeq Nano DNA Library Preparation Kit (Illumina, San Diego, CA) according to the manufacturers instructions with a final average library size distribution (including adapters) of 650 bp. Sequencing was done using Illumina Hi-Seq 2500 (one lane) to generate a total of 83,595,840 100 bp paired-end reads with an insert size of ~600 bp. Data was processed using RTA v1.18.64, with default filter and quality settings. The reads were demultiplexed with CASAVA v1.8.4 (allowing no mismatch).

### Assembly

Before genome analysis could be carried out it was necessary to separate the cyanobacterial genome from non-cyanobacterial sequences. An ideal prokaryotic assembly produced by a de Bruijn graph based assembler such as SPAdes [37] would produce a single chromosome where there is only one possible path between nodes (i.e., individual contigs). However, ambiguities often remain in an assembly (e.g., due to repeated regions or duplicated genes) that result in de Bruijn graphs with multiple edges (i.e., possible alignments between contigs). While these multi-edges prevent the formation of a single conclusive chromosome, they can be used to determine relationships between clusters of smaller contigs. While this information is usually discarded it can be used to isolate individual genomes from a metagenome, and validated where contigs can be simultaneously defined by some other shared characteristic such as read depth, BLAST similarity or sequence composition. Visualisation of this information has recently been made available in the de Bruijn graph viewer Bandage [38].

Prior to assembly Illumina TruSeq-3-PE adapters were trimmed and reads filtered for quality using Trimmomatic v0.32 [39] using the following parameters: Leading:20, Trailing:20, SlidingWindow:4:20, MinLen:50. Error correction and assembly was done using SPAdes v3.5.0 [37] with k-mer lengths of 67, 77, 87 and 97 and a coverage cut-off of 20. SPAdes FASTG files were then opened in Bandage v0.07 [38] and a BLAST database generated for the entire assembly. One thousand and fifty-four core cyanobacterial clusters of orthologous groups of proteins (core CyOGs) (see Mulkidjanian et al. [40], Supplementary Information) exclusive to cyanobacteria and plastids were searched against this database using tBLASTn v2.2.30+ with an e-value threshold of 1e-10. Cyanobacterial contigs were identified as graph nodes containing core CyOGs and connected nodes with similar read depth. Additional unconnected nodes with similar coverage to confirmed cyanobacterial contigs were checked manually by performing further tBLASTn searches against the entire NCBI GenBank database (e-value threshold of 1e-10); those that did not contain putative cyanobacterial genes were discarded. Finally, contigs with read depth <10 were discarded to remove remaining non-cyanobacterial contigs and contigs with length <200 bp were discarded to bring into line with NCBI standards. Raw reads were mapped to the resulting draft assembly using BWA [41]. Reads that did not map to the cyanobacterial assembly were discarded, revealing an overall coverage of the *P. priestleyi* BC1401 genome of 340.55×.

The draft genome was submitted to JGI IMG/ER [42] for annotation (GOLD Analysis Project ID: Ga0078185). Based on sequence similarity, the assembled genome was aligned to its nearest neighbour with a finished genome, *Leptolygnbya boryana* PCC 6306 with Contiguator v2.7.4 [43] using BLASTn with an e-value of 1e-10, a contig length threshold of 1000, a hit length threshold of 1000 and a contig coverage threshold of 10 %. Identification of specific genes of interest was done using BLASTp (e-value threshold of 1e-5) and further searches for Pfam domains were made if BLAST searches yielded ambiguous results. These analyses were performed using tools available in JGI IMG/ER [42].

To infer structural homology of proteins where certain domains were absent, genes were aligned in Jalview v2.9.0b2 [44] and protein structures modelled using the Phyre2 web portal [45].

Genome plots were produced in Circos v0.68 [46] and gene diagrams were made using FancyGene v1.4 [47]. All plots were manually edited using Inkscape v0.91 [48].

This Whole Genome Shotgun project has been deposited at DDBJ/ENA/GenBank under the accession LXYR00000000. The version described in this paper is version LXYR01000000. Genome sequence and annotation data are available at the JGI IMG/ER database [49].

### Phylogenetic analysis

A SSU rRNA dataset was constructed including *P. priestleyi* BC1401 and a further 130 cyanobacteria genomes as used by Sánchez-Baracaldo [50]. Additionally, the SSU rRNA sequence *P. priestleyi* ANT.L61.2 [51] was included as a reference for an Antarctic strain of *P. priestleyi*. An alignment of 1,712 characters was generated using SATé 2.2.7 [52] (using MAFFT v6.717 [53], MUSCLE v3.7 [54], FASTTREE v2.1.4 [55] with the CAT approximation and decomposition strategy set to ‘longest’). Phylogenetic reconstruction was carried out using RaxML v8.1.11 [56] using the GTR + G model. The tree was constrained using the 130 taxa phylogenomic (135 protein and two rRNA: LSU and SSU) tree described in Sánchez-Baracaldo [50]. Trees were visualised using FigTree v1.4.0 [57] and manually edited in Inkscape v0.91 [48].

## Results and discussion

**Fig. 1:**
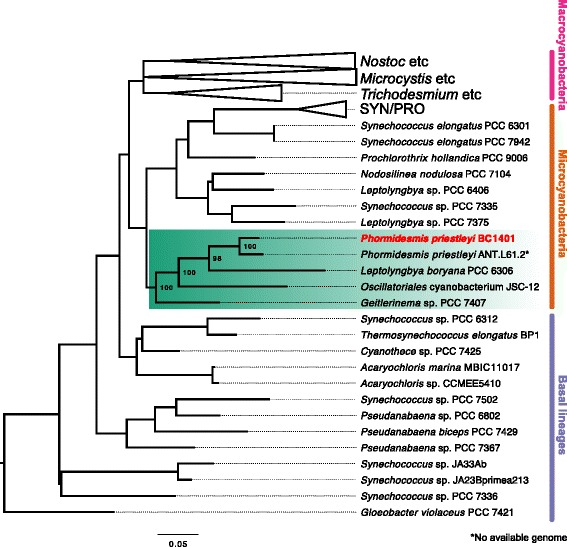
SSU rRNA maximum likelihood phylogeny showing relatives of *P. priestleyi* BC1401. Tree topology was enforced using a phylogenomic constraint tree containing 130 taxa (135 protein and two rRNA: LSU and SSU) [50]. The tree is divided into ‘Basal lineages’, ‘Microcyanobacteria’ and ‘Macrocyanobacteria’ as described in Sánchez-Baracaldo [50]. The clade containing *P. priestleyi* BC1401 and related lineages is highlighted in green with bootstrap support (1000 replicates) shown

Our phylogenetic analyses indicate that *P. priestleyi* BC1401 is sister to *Phormidesmis* ANT.L61.2 from the McMurdo Dry Valleys, Antarctica. These two lineages are sister to *L. boryana* PCC 6306, Oscillatoriales cyanobacterium JSC-12 and *Geitlerinema* sp. PCC 7407 (Fig. 1). These strains are nested within a group of small cell diameter filamentous lineages within the Microcyanobacteria [50]. Genome statistics and sampling locations for all complete genomes used in this study are shown in Table 1.

**Table 1:** Genome characteristics of strains used in this study

	*Phormidesmis priestleyi* BC1401	*Leptolyngbya boryana* PCC 6306	Oscillatoriales cyanobacterium JSC-12	*Geitlerinema* sp. PCC 7407
Geographic location	Greenland Ice Sheet	Unknown	Yellowstone, USA	Unknown
Habitat	Cryoconite	Temperate lake	Hot spring	Unknown
Environmental temperature	~0–3 °C	~4–20 °C	~37–54 °C	Unknown
Genome size(bp)	5,551,896	7,262,454	5,530,391	4,681,111
DNA coding (%)	83.8	87.93	86.41	84.37
DNA G+C (%)	49.16	47.01	47.47	58.46
DNA scaffolds	213	5	1	1
Scaffold size (bp)	213–275,582	27,440–6,255,543	5,530,391	4,681,111
N50	79,760	6,255,543	5,530,391	4,681,111
Total genes	5,612	6,911	5,081	3,913
PEGs	5,507	6,827	5,024	3,854

**Fig. 2:**
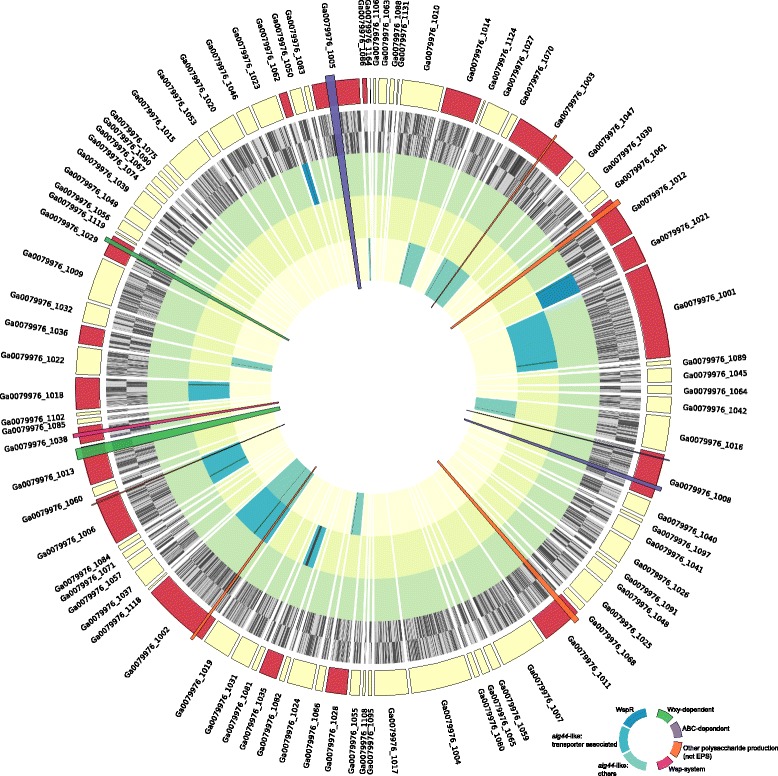
Circular plot of the *P. priestleyi* BC1401 genome ordered according to *L. boryana* PCC 6306. Rings are as follows (outer - inner): 1) Mapped contigs, contigs containing genes putatively involved in EPS biosynthesis and export are highlighted in *red*; 2) Annotated genes are shown in dark grey, outer = plus strand, inner = minus strand; 3) putative *wspR* homologues are shown in dark grey, contigs are highlighted in *blue*; 4) *alg44*-like genes that are components of ABC-transporters are shown in dark grey, contigs are highlighted in *blue*; 5) other *alg44*-like genes are shown in dark grey, contigs are highlighted in *blue*. Wedges show locations of putative gene clusters involved in Wzy-, ABC-dependent-, Wsp- and other non EPS specific polysaccharide biosynthesis/export pathways

The de Bruijn graph of the entire raw assembly consisted of 752 nodes with 439 edges and a total length of 21,126,715 bp representing a metagenome containing both *P. priestleyi* BC1401 and its associated microbiota. The main cyanobacterial portion of the graph was determined as a cluster of 268 nodes linked by 355 edges (5,524,226 bp) with a mean depth of 16.5. Of these, 49 nodes with 3 edges (3,795,178 bp) and a mean depth of 14.1 had positive BLAST hits for core CyOGs. All BLAST hits for core CyOGs were contained within the main cyanobacterial portion of the graph [see Additional file 1]. The final assembled draft genome of *P. priestleyi* BC1401 constituted a total of 213 contigs containing 5,507 protein-encoding genes (PEGs). Contigs ranged from 213 to 2,755,82 bp and the assembly had an N50 of 79,760 bases. Genome size was estimated to be 5.55 Mb with an overall GC content of 49.16 %. The sequenced genome size of *P. priestleyi* BC1401 was found to be smaller than that of its closet relative with a complete genome, (SSU rRNA gene identity = 92 %) *L. boryana* PCC 6306 (size = 7.26 Mb, GC = 47.01 %), while having slightly higher GC content. Despite the smaller size in relation to *L. boryana* PCC 6306 all 1,054 core CyOGs were present in the *P. priestleyi* BC1401 draft genome suggesting a nearly complete assembly. Eighty-five contigs were successfully mapped to *L. boryana* PCC 6306 (4.77 Mb, 85.97 % of the total draft genome) (Fig. 2) leaving 128 smaller contigs (0.78 Mb, 14.03 % of the total draft genome) unmapped. A plot displaying all unmapped contigs can be seen in Additional file 2. Forty-five of these contigs were below 1,000 bp in length and 77 were dropped from the alignment due to low coverage.

### Cold adaptation

Molecular evolution related to cold adaptation can leave distinct signatures on organism’s genomes (e.g., Bacteria: *Psychrobacter arcticus* 273–4 [58], *Colwellia psychrerythraea* 34H [59]; Archaea: *Halobrum lacusprofundi* [60], *Methanogenium frigidum* and *Methanococcoides burtonii* [61]. In particular, the tendency of certain amino acids to influence protein structures at low temperatures can lead to the substitution of one amino acid for another. For example the amino acid arginine readily forms H-bonds in protein secondary structures; in low temperature environments this can inhibit protein flexibility and prevent optimal protein function. As such, cold adapted organisms may substitute arginine residues with lysine to reduce structural stability at low temperatures as seen in *Alteromonas haloplanctis* [62]. Similarly, a reduction in proline content can also increase the flexibility of a protein’s secondary structure [63, 64] (e.g., *Psychrobacter immobilis* A5 [65]). The percentage of proline residues in the hypothetical proteome of *P. priestleyi* BC1401 (Pro% = 4.76) was similar to that of L. *boryana* PCC 6306 (Pro% = 4.66) and Oscillatoriales cyanobacterium JSC-12 (Pro% = 4.78) and was slightly higher in *Geitlerinema* sp. PCC 7407 (Pro% = 5.44). The ratio of arginine to lysine in BC1401 (Arg:Lys = 1.4) was also comparable to PCC 6306 (Arg:Lys = 1.45) and JSC-12 (Arg:Lys = 1.49), while being slightly higher in PCC 7404 (Arg:Lys = 2.22). Given the large temperature ranges experienced by each of these strains (Table 1) it appears that there is no obvious signal of increased genomic adaptation to the cold in BC1401 compared to other cyanobacteria typically found in warmer habitats.

Temperature stress in bacteria is managed by cold shock or heat shock mechanisms, allowing cells to function at low temperatures or to survive above their thermal optimum. In bacteria, many genes have been identified that encode proteins related to cellular responses to cold stress (e.g., pyruvate dehydrogenases, DNA gyrases, chaperones and fatty acid desaturases) [5, 66]. Several of the proteins implicated in cold shock also act as heat shock proteins. Since the physiological optimum of cryoconite phototrophs is above that encountered in the environment [67], cold stress is likely very important in *P. priestleyi* BC1401. However, if organisms are acclimated to growing at low temperatures then in the event of sudden warming a heat stress response may still occur. The numbers of BLAST hits in the *P. priestleyi* BC1401 genome for genes implicated in cold shock response as compared to its three closest relatives with complete genomes (*Leptolyngbya boryana* PCC 6306, Oscillatoriales cyanobacterium JSC-12 and *Geitlerinema* sp. PCC 7407) are shown in Table 2. All genes listed were present in all genomes with the exception of the following: *csp*-family genes were absent from Oscillatoriales cyanobacterium JSC-12, *ots*A was found only in *Geitlerinema* sp. PCC7407, and *yfiaA* was absent from *L. boryana* PCC6306. Otherwise, copy number variation between the four genomes was minimal with no clear increase in cold stress genes in *P. priestleyi* BC1401. This included genes for chaperones that are also involved in heat shock response (*dnaK*, *dnaJ*) and no difference in copy number among the compared cyanobacterial genomes was found for the heat shock specific gene *grpE* [68]. Even so, it is possible that currently uncharacterised genes involved in cold tolerance (e.g. production of ice nucleation proteins or resistance to osmotic stress [69]) may yet remain unidentified within the *P. priestleyi* BC1401 genome.

**Table 2:** Numbers of BLAST hits for genes implicated in bacterial cold shock response in all strains used in this study

Gene	Number of BLAST hits			
	*Phormidesmis priestleyi* BC1401	*Leptolyngbya boryana* PCC 6306	Oscillatoriales cyanobacterium JSC12	*Geitlerinema* sp. PCC7407
*aceE*	1	2	2	1
*aceF*	1	2	1	1
*csp*-family	1	1	0	1
*deaD*	1	1	1	1
*desAB*	2	2	1	2
*dnaA*	1	1	1	1
*gyrA*	2	2	2	2
*dnaK* ^a^	5	5	6	4
*dnaJ* ^a^	1	1	1	1
*hupB*	4	3	3	1
*infA*/IF-1	1	1	1	1
*infB*/IF-2	1	1	1	1
*infC*/IF-3	1	1	2	2
*nusA*	1	1	1	1
*otsA*	0	0	0	1
*pnp*	1	1	1	1
*rnr*	1	1	1	1
*rbfA*	1	1	1	1
*recA*	1	1	1	1
*tig*	1	1	1	1
*yfiA*	1	0	1	1
Total	29	29	29	27

Compiled from Barria et al. [5] and Varin et al. [66]. Chaperones also involved in heat shock are marked with ^a^

The apparent absence of differentiation of cold shock genes between *P. priestleyi* BC1401 and temperate lineages could be related to the tendency of polar cyanobacteria to be psycrotrophs rather than psychrophiles, exhibiting optimal growth at far higher temperatures than the low ambient temperatures that they are likely to experience in the environment [70]. In light of this, it may be that more universal mechanisms such as the production of EPSs are responsible for the success of cyanobacteria in cold environments.

### EPS production mechanisms in *P. priestleyi* BC1401

While not fully characterised experimentally in cyanobacteria, the genes responsible for EPS biosynthesis and export in cyanobacteria have been clearly outlined in Pereira et al. [71]. These genes encode the constituent proteins of several specific pathways in gram-negative bacteria that were initially studied in *E. coli* (reviewed in Cuthbertson et al. [72]). In the following sections, we report the distribution of these genes throughout the genome of *P. priestleyi* BC1401.

Production of prokaryotic EPSs within the cell typically occurs in three main steps [15]. First, oligosaccharides are synthesised within the cytoplasm. Second, repeating units of oligosaccharides are assembled and attached to a lipid carrier by glycosyltransferases. Finally, repeating units are polymerised and translocated out of the cell. This final step is carried out by one of three main systems: the Wzy-dependent, ATP-binding cassette (ABC)-dependent and synthase dependent pathways [16, 64, 73].

**Fig. 3:**
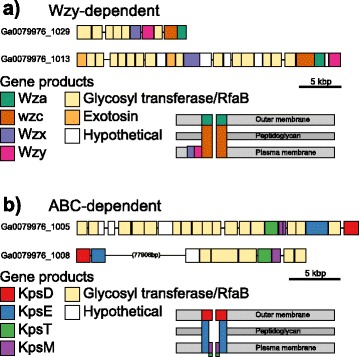
Gene diagrams of putative **a**) Wzy- and **b**) ABC-dependent clusters in *P. priestleyi* BC1401. Schematics of the organisation of proteins within the cell membrane are shown (based on Pereira et al. [71])

In the Wzy-dependent pathway, lipid linked oligosaccharide units are translocated from the cytoplasm to the periplasm by Wzx where they are polymerised by Wzy. Export then occurs via the transmembrane polysaccharide co-polymerase (PCP) Wzc and outer membrane transporter (OPX) Wza. Two gene clusters in *P. priestleyi* BC1401 followed the scheme for a Wzy-dependent EPS export system, one on contig Ga0079976_1029 and a second on Ga0079976_1013 (Fig. 3a). Both clusters included genes containing domains that are conserved in the genes *wza, wzc, wzx*, and *wzy*. In both clusters, *wza* and *wzc* were adjacent to each other, while the arrangement of the remainder of associated genes differed considerably. Both clusters included genes containing domains involved in biosynthesis (WcaA domains) and assembly (glycosyltransferases, RfaB domains). Two genes in the cluster on contig Ga0079976_1013 are putatively annotated as exotosins, the glucuronosyltransferase domain of which has been shown to be homologous to the *mur*3 gene in *Arabidopsis thaliana* [74]. *mur*3 is responsible for modification of xyloglucans (involved in crosslinking of cellulose matrices) and the genes found here may encode proteins responsible for similar functions that contribute to the structure of the EPS matrix.

In the ABC-dependent pathway, polymerisation occurs in the cytoplasm and the ABC-transporter KpsTM transfers assembled polysaccharides across the cytoplasmic membrane, before they are exported from the cell by the PCP/OPX proteins KpsD and KpsE. Two clusters in *P. priestleyi* BC1401 contained components for the ABC-dependent pathway (Fig. 3b). Gene clusters on contigs Ga0079976_1005 and Ga0079976_1008 contain genes with domains found in *kpsD*, *kpsE*, *kpsM* and *kpsT*. On contig Ga0079976_1005, *kpsM* was annotated as two smaller genes and all of the *kps*_ genes were clustered close together, whereas on Ga0079976_1008 *kpsD* and *kpsE* were located ~80kbp upstream of the *kpsM* and *kpsT* homologues. As with the Wzy-dependent clusters, several biosynthetic and assembly genes were also present, suggesting relatively self-contained modules for EPS synthesis and export in *P. priestleyi* BC1401.

In the synthase-dependent pathway, a single protein, Alg8 (which is regulated by Alg44) carries out synthesis, polymerisation and translocation from the cytoplasm to the periplasm. Export is via an outer membrane porin, AlgE which is scaffolded to the membrane by AlgK. Despite several *alg8* homologs being found in the genome, no clear synthase-dependent pathways were identified in *P. priestleyi* BC1401. This is consistent with the findings of Peireira et al. [71] who were unable to confidently identify key components of the synthase-dependent pathway throughout the entire phylum of cyanobacteria.

**Fig. 4:**
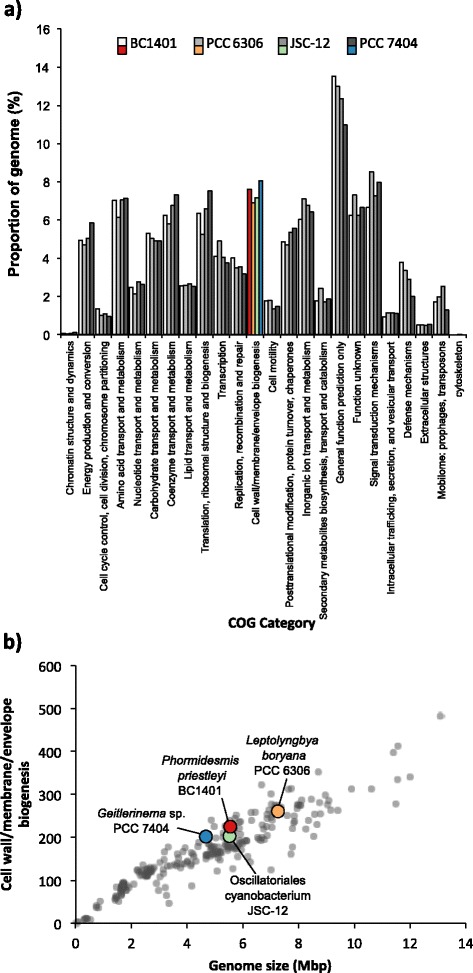
Plots showing proportion of genes involved in COG category ‘Cell wall/membrane/envelope biogenesis’ in *P. priestleyi* BC1401, *L. boryana* PCC 6306, Oscillatoriales cyanobacterium JSC-12 and *Geitlerinema* sp. PCC 7407. **a** Proportion of all COGs, ‘Cell wall/membrane/envelope biogenesis’ is highlighted and **b**) The number of ‘Cell wall/membrane/envelope biogenesis’ genes compared to genome size in the context of 265 other cyanobacterial genomes publicly available on JGI IMG/ER

Out of the known prokaryotic EPS export pathways *P. priestleyi* BC1401 contained two Wzy- and two ABC-dependent gene clusters and no complete synthase dependent gene clusters, similar to many other cyanobacteria irrespective of the environment [71]. Despite the importance of EPS production for survival in cold environments, there was no indication that the genome of *P. priestleyi* BC1401 had any relative increase in EPS related genes. EPS genes are contained within the cluster of orthologous genes (COG) category ‘Cell wall/membrane/envelope biogenesis’. A total of 225 genes representing 7.61 % of the *P. priestleyi* BC1401 genome were included in the ‘Cell wall/membrane/envelope biogenesis’ category (see Fig. 4a) compared to 6.91 % in *L. boryana* PCC 6306, 7.17 % in Oscillatoriales cyanobacterium JSC-12 and 8.05 % in *Geitlerinema* sp. PCC 7407. Peireira et al. [71] indicated that genome size rather than habitat had the largest effect on number of EPS related genes. *P. priestleyi* BC1401 appears to be no exception to this with the number of ‘Cell wall/membrane/envelope biogenesis’ genes falling well within the expected distribution of genomes that size (see Fig. 4b).

### Environmental sensing

**Fig. 5:**
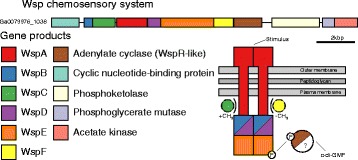
Gene diagrams of putative Wsp-chemosensory system in *P. priestley*i BC1401. Schematics of the organisation of proteins within the cell membrane are shown (based on Belas [79])

**Fig. 6:**
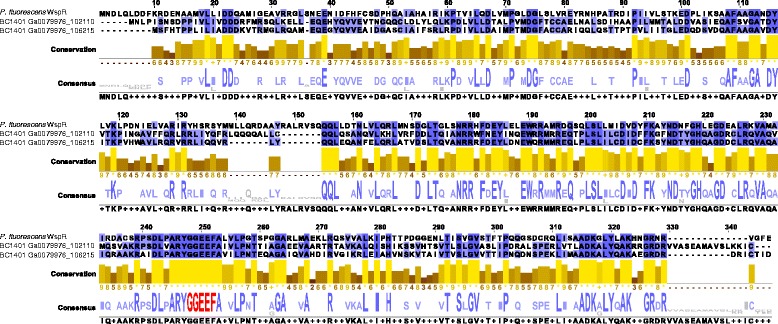
Alignment of two putative WspR homologues in *P. priestleyi* BC1401 with the WspR of *Pseudomonas fluorescens*. The GGDEF/GGEEF domain is highlighted in *red*

While it is clear that many cyanobacteria produce EPSs, the way in which EPS production is regulated in cyanobacteria is not yet understood. The bacterial second messenger bis (3′-5′)-cyclic dimeric guanosine monophosphate (c-di-GMP) has been implicated in a large number of signalling pathways related to EPS biosynthesis and biofilm formation [75, 76]. It is synthesised by proteins containing the conserved GGDEF/GGEEF domain and binds to a wide variety of cellular receptors, which are normally proteins containing PilZ domains. Synthesis of c-di-GMP can be regulated by the Wsp system (first identified in *Pseudomonas fluorescens*), a chemosensory pathway thought to modulate c-di-GMP levels in response to surface adhesion causing biofilm formation and a transition from motile to sessile lifestyle [77–79]. A nearly complete cassette for the Wsp chemosensory pathway was found in *P. priestleyi* BC1401, located on contig Ga0079976_1038 (Fig. 5). A gene for a cyclic nucleotide binding protein is followed on the forward strand by *wspB*, *wspC*, *wspA*, *wspD*, *wspE*, *wspF*. In the position normally occupied by the response regulator *WspR* was a class 3 adenylate cyaclase. Whereas WspR is a GGDEF/GGEEF domain containing diguanylate cyclase, class 3 adenylate cyclases are cyclase homology domain containing mononucleotidyl cyclases. Searches for GGDEF/GGEEF pfam domains within the *P. priestleyi* BC1401 genome revealed six potential *wspR* homologues. In-silico modelling of their structures using Phyre2 revealed two proteins featuring homology to WspR with 100 % confidence over an alignment length of >90 % (Table 3, Fig. 6). One of these is a single gene on contig Ga0079976_1021 and the second is on contig Ga0079976_1062. A further five genes contained GGDEF/GGEEF domains but showed low sequence similarity for the remainder of the protein (Table 3).

**Table 3:** Locations of genes with GGDEF/GGEEF domains in the *P. priestleyi* BC1401 draft genome and similarity of the gene product to WspR based upon modelling of protein structure in Phyre2 [45]

BC1401 gene	Gene Product Name	Length (aa)	Confidence	%ID	Alignment length
Ga0079976_102110	Response regulator receiver modulated diguanylate cyclase^a^	331	100 %	38 %	93 %
Ga0079976_106215	Response regulator receiver modulated diguanylate cyclase^a^	312	100 %	39 %	97 %
Ga0079976_102431	Response regulator receiver modulated diguanylate cyclase/phosphodiesterase	595	100 %	27 %	50 %
Ga0079976_101496	Response regulator receiver modulated diguanylate cyclase/phosphodiesterase	613	100 %	27 %	51 %
Ga0079976_10199	PAS domain S-box-containing protein/diguanylate cyclase (GGDEF) domain-containing protein	725	100 %	29 %	23 %
Ga0079976_10297	Diguanylate cyclase (GGDEF) domain-containing protein	530	100 %	34 %	21 %
Ga0079976_101051	PAS domain S-box-containing protein/diguanylate cyclase (GGDEF) domain-containing protein	732	100 %	34 %	22 %

Putative *wspR* homologues are marked with ^a^

For a standard WspR regulated Wsp-system to influence EPS production and biofilm formation, a receptor must exist for synthesised c-di-GMP. Somewhat unexpectedly, genes containing the typical c-di-GMP binding PilZ domains were apparently absent from the *P. priestleyi* BC1401 draft genome. Blast searches for homologues of the gene for Alg44, the c-di-GMP binding component of the absent synthase dependent pathway, revealed 14 genes that shared the HlyD domain of *alg44* while the PilZ domain appeared to be missing (Table 4). Five of these were clustered together with ABC-transporter genes; one such cluster was homologous to DevBCA, the efflux transporter responsible for formation of the heterocyst envelope in diazotrophic cyanobacteria [80, 81]. It should be stressed that ABC-transporters are involved in a wide variety of cellular processes and are not necessarily linked to EPS. Other genes have also been implicated in c-di-GMP binding. For example, non-enzymatic GGDEF/GGEEF domains (e.g., PopA and PelD) have also been shown to operate as c-di-GMP effectors [72]. At least one such protein may exist in *P. priestleyi* BC1401 where a GGDEF/GGEEF domain was identified but the GGDEF/GGEEF motif itself was absent (Ga0079976_10297). However, despite the clear existence of a well-defined Wsp-like chemosensory system, the exact nature of the system’s response regulators and effectors remain unclear. The close BLAST similarity of Wsp genes in *P. priestleyi* BC1401 to those in several other cyanobacteria suggest that this may be an important pathway across the cyanobacteria and warrants further investigation if we are to understand the way in which cyanobacteria interact with their environment.

**Table 4:** Locations of putative *alg44* homologues lacking PilZ domains in the *P. priestleyi* BC1401 draft genome

BC1401 Gene	Gene product name	Length (aa)	Domains
Ga0079976_1001251	HlyD family secretion protein^a^	466	Biotin_lipoyl_2(pfam13533)HlyD_D23(pfam16576)
Ga0079976_100279	HlyD family secretion protein^ab^	395	HlyD_3(pfam13437) Biotin_lipoyl_2(pfam13533)
Ga0079976_100288	HlyD family secretion protein	477	HlyD_3(pfam13437) Biotin_lipoyl_2(pfam13533)
Ga0079976_100319	RND family efflux transporter, MFP subunit	484	HlyD_D23(pfam16576)
Ga0079976_100646	HlyD family secretion protein^a^	385	HlyD_3(pfam13437) Biotin_lipoyl_2(pfam13533)
Ga0079976_10149	membrane fusion protein, multidrug efflux system	463	Biotin_lipoyl_2(pfam13533) HlyD_D23(pfam16576)
Ga0079976_101677	RND family efflux transporter, MFP subunit	480	HlyD_D23(pfam16576)
Ga0079976_101876	HlyD family secretion protein^a^	438	Biotin_lipoyl_2(pfam13533)HlyD_D23(pfam16576)
Ga0079976_102849	membrane fusion protein, multidrug efflux system	556	HlyD_D23(pfam16576)
Ga0079976_103537	HlyD family secretion protein^a^	434	HlyD_3(pfam13437) Biotin_lipoyl_2(pfam13533)
Ga0079976_103615	RND family efflux transporter, MFP subunit	438	Biotin_lipoyl_2(pfam13533)HlyD_D23(pfam16576)
Ga0079976_104331	HlyD family secretion protein	509	HlyD_3(pfam13437) Biotin_lipoyl_2(pfam13533)
Ga0079976_106912	RND family efflux transporter, MFP subunit	443	HlyD_3(pfam13437) Biotin_lipoyl_2(pfam13533)
Ga0079976_10868	membrane fusion protein, cobalt-zinc-cadmium efflux	530	HlyD_D23(pfam16576)

Genes associated with ABC-transport genes are marked ^a^. Genes associated with DevBCA-type exporters are marked^b^

The mechanisms of EPS production and regulation described here are by no means unique to *P. priestleyi* BC1401 and can be found throughout the cyanobacterial phylum [71]. However, they are also not ubiquitous and lineage specific characteristics may mean that certain cyanobacteria are predisposed to exploit certain environments or interact with their environment in particular ways. For example, the Wsp system is not present in all cyanobacteria, being identified in *L. boryana* PCC 6306 and *Geitlerinema* sp. PCC 7407 but not in Oscillatoriales cyanobacterium JSC-12. This implies that closely related lineages may interact with their environment in very different ways. In terms of *P. priestleyi* BC1401, the presence of the Wsp system may have considerable implications for the mechanisms of cryoconite formation. Physical contact of the cell surface of *P. priestleyi* with particulate matter may result in the activation of biofilm formation and EPS production mechanisms, thus initiating the first stages of cryoconite aggregation. Further investigation into the regulation and expression of this and the EPS production mechanisms will help us to understand how cyanobacteria influence ice sheet surfaces.

## Conclusions

The work presented here represents a first step in understanding the molecular underpinnings of adaptation of cyanobacteria to cold environments and raises many questions. The Arctic cyanobacterium *P. priestleyi* BC1401 is closely related to the Antarctic *P. priestleyi* based upon SSU similarity and likely represents a cryosphere specific lineage. A standard complement of cold shock genes and a lack of cold biased molecular evolution, as has been seen in other prokaryotes, suggests that *P. priestleyi* BC1401 does not require these characteristics to tolerate survival in cold environments. Instead, it is likely that the production of EPSs buffers it from the extreme conditions that it experiences. Why *P. priestleyi* is found in the cryosphere while closely related lineages bearing similar genome characteristics are not is not yet known. This is intriguing since the EPS synthesis and export mechanisms in *P. priestleyi* BC1401 appear to follow the same scheme that has been reported from throughout the cyanobacterial phylum. Any differences may then be a result of regulation and differential expression under the conditions experienced in cold environments. As a result, future work should include targeted transcriptomics to understand how these genes are expressed and what implications that may have for both the organism and the environment it inhabits. Furthermore, the Wsp chemosensory pathway represents a possible link between the environment and production of EPS and understanding how this operates in *P. priestleyi* BC1401 may help us to better understand the principles of cryoconite formation.

## Abbreviations

ABC, ATP-binding cassette; AF, alignment fraction; C-di-GMP, bis (3′-5′)-cyclic dimeric guanosine monophosphate; COG, clusters of orthologous genes; CyOG, cyanobacterial clusters of orthologous groups of proteins; EPS, exopolysaccharide; gANI, genomic average nucleotide identity; GrIS, Greenland Ice Sheet; MAA, mycosporine-like amino acid; OPX, outer membrane transporter; PCP, polysaccharide co-polymerase; PEG, protein-encoding genes; SSU, small subunit

## Additional files

Additional file 1: Visualisation of the assembled *P. priestleyi* BC1401 metagenome. Output from Bandage v0.07 [38]. Contigs with a depth <10 are shown in blue, contigs with a depth >10 are shown in orange. Positive BLAST hits (minimum e-value = 1e-10) for core CyOGs as determined by Mulkidjanian et al. [40] are indicated with overlapping labels. All of the contigs containing core CyOGs and the majority of contigs with coverage >10 are all contained within a single subgraph. (PDF 1463 kb) Additional file 2: Circos plot showing *P. priestleyi* BC1401 contigs that did not map to *L. boryana* PCC6306. Contigs are ordered according to size in an anticlockwise direction. (PDF 20 kb)
