# Supplementary figures and images for: Genomic mechanisms for cold tolerance and production of exopolysaccharides in the Arctic cyanobacterium Phormidesmis priestleyi BC1401
Source: BMC Genomics. 2016 Aug 2;17:533. doi: 10.1186/s12864-016-2846-4 (PMC4971617; doi:10.1186/s12864-016-2846-4)

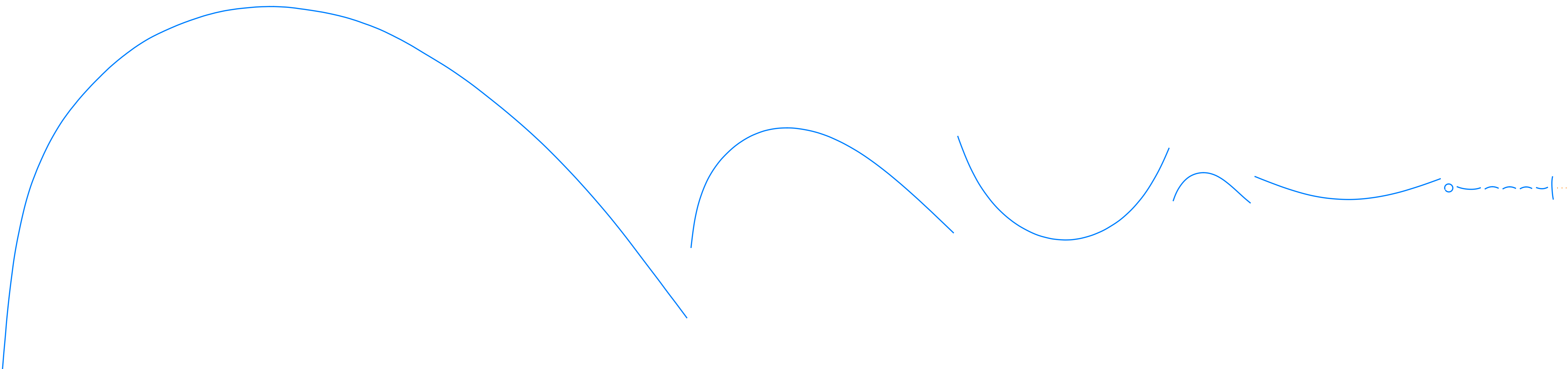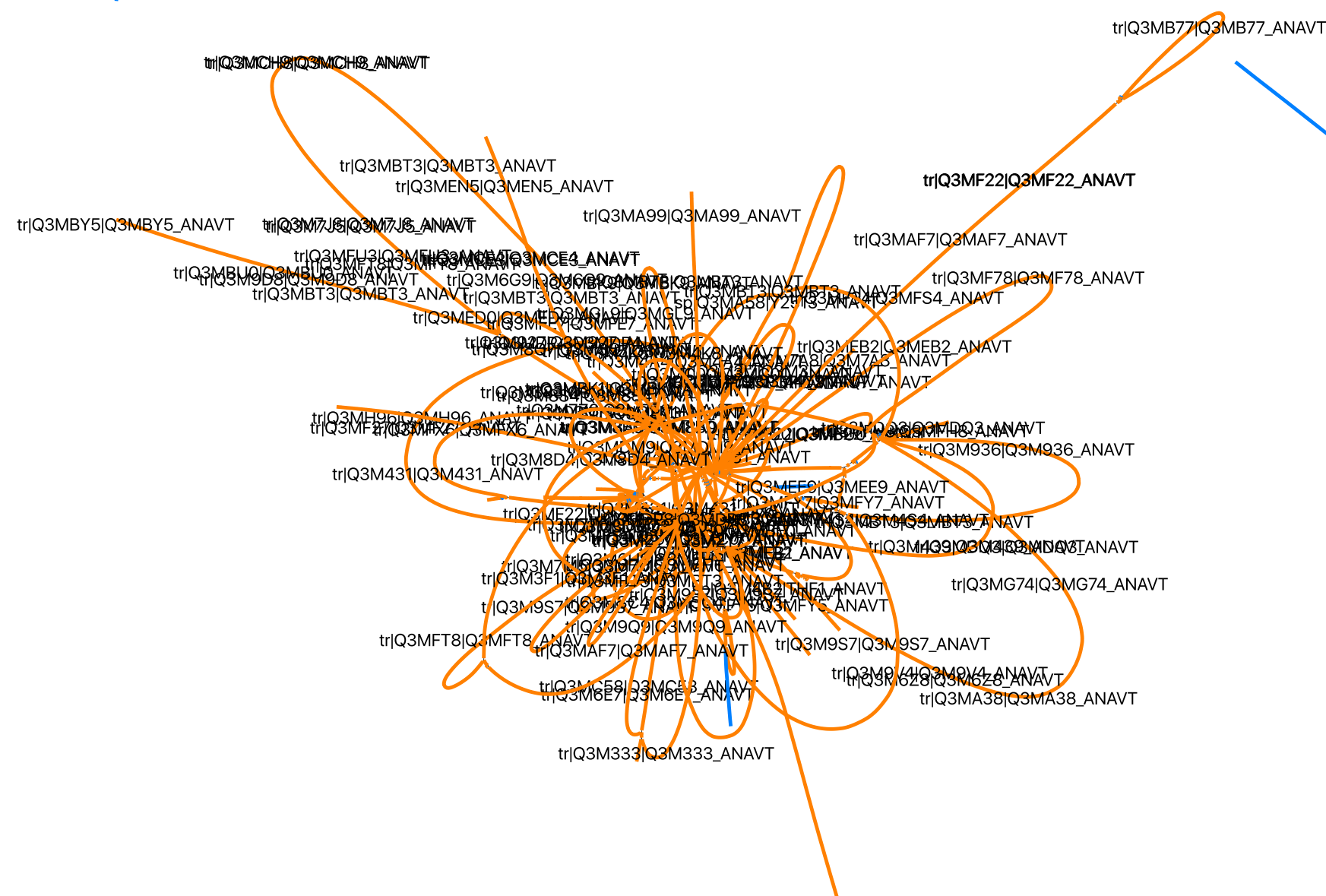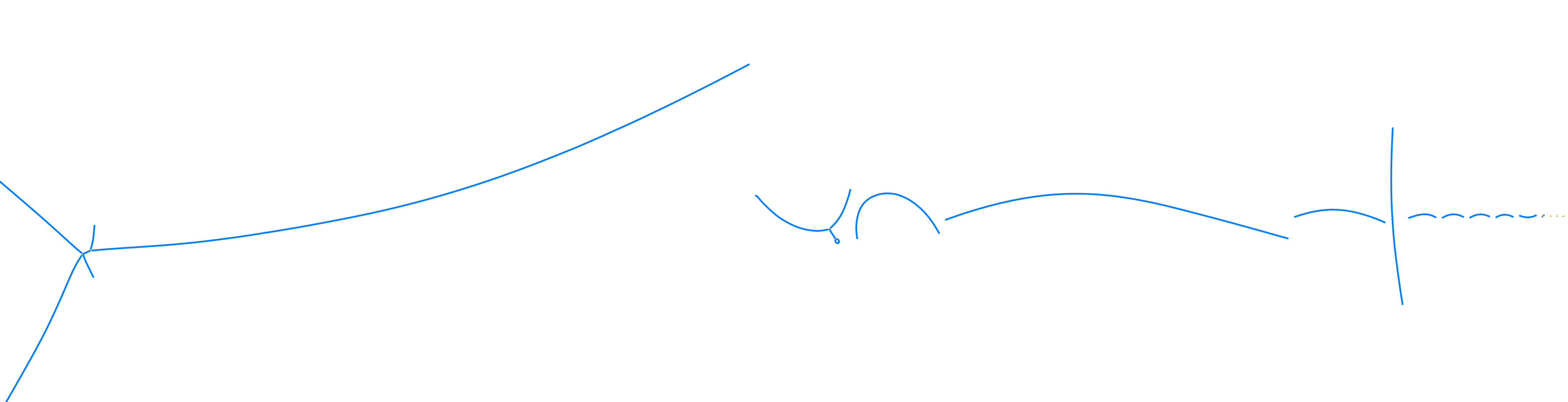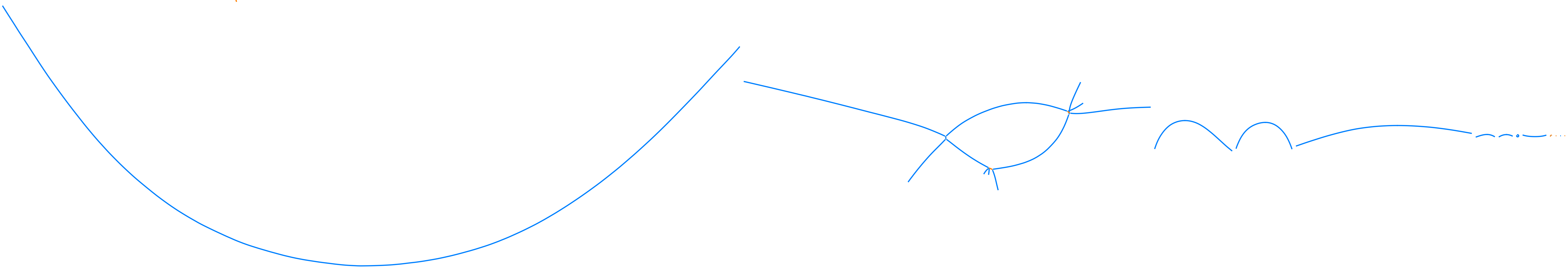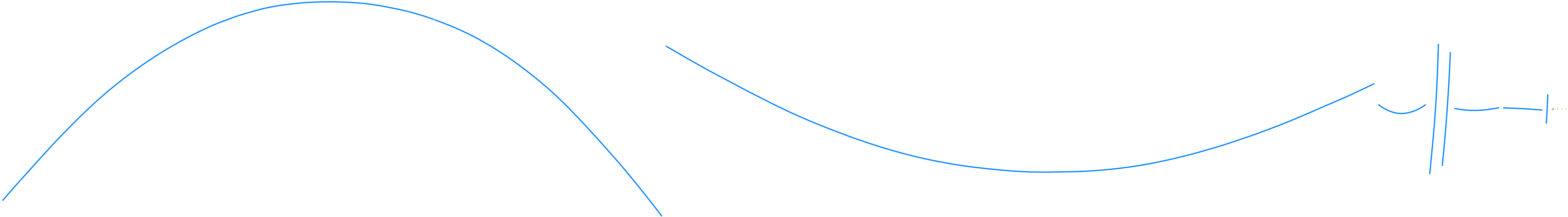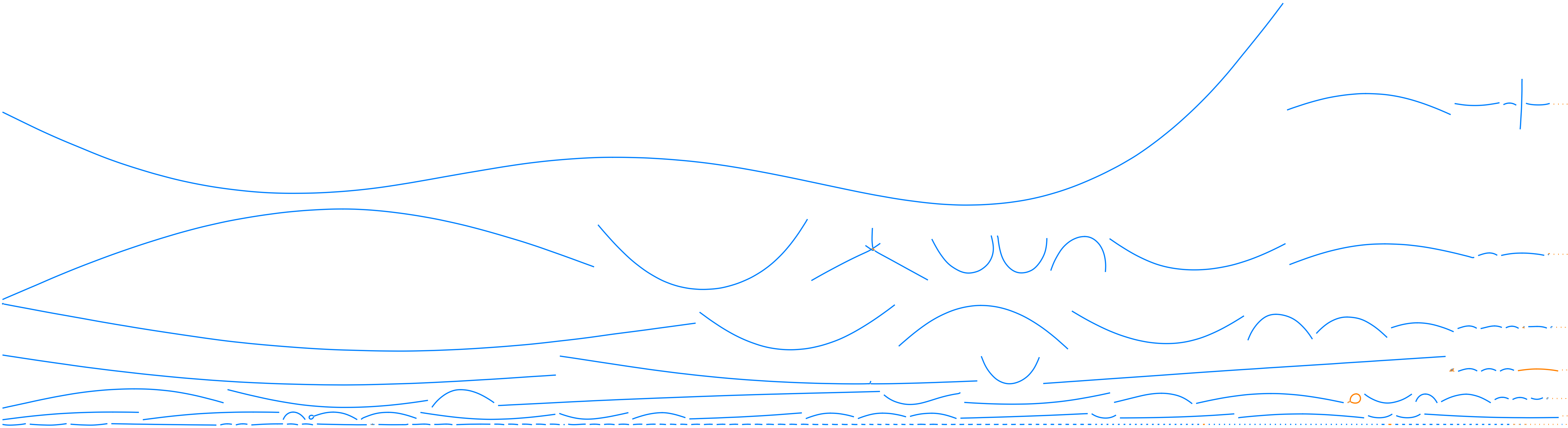

Supplement: Additional file 1: — Visualisation of the assembled P. priestleyi BC1401 metagenome. Output from Bandage v0.07 [38]. Contigs with a depth <10 are shown in blue, contigs with a depth >10 are shown in orange. Positive BLAST hits (minimum e-value = 1e-10) for core CyOGs as determined by Mulkidjanian et al. [40] are indicated with overlapping labels. All of the contigs containing core CyOGs and the majority of contigs with coverage >10 are all contained within a single subgraph. (PDF 1463 kb) [file 12864_2016_2846_MOESM1_ESM.pdf]

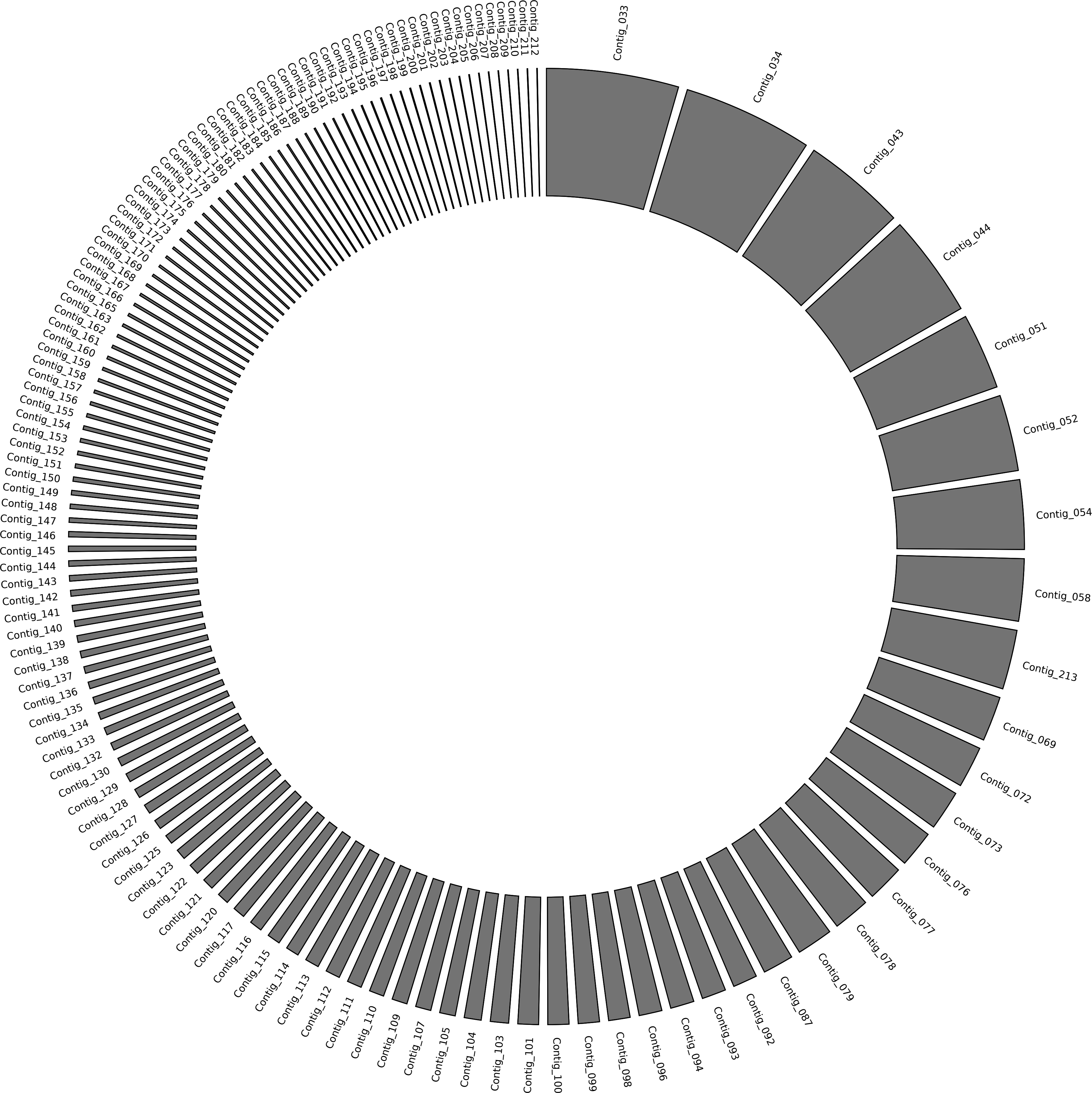

Supplement: Additional file 2: — Circos plot showing P. priestleyi BC1401 contigs that did not map to L. boryana PCC6306. Contigs are ordered according to size in an anticlockwise direction. (PDF 20 kb) [file 12864_2016_2846_MOESM2_ESM.pdf]
